# Vaginal microbiome dysbiosis and a rectal reservoir of uropathogens characterize postmenopausal women with recurrent urinary tract infections: a cross-sectional study

**DOI:** 10.3389/fmicb.2026.1812000

**Published:** 2026-04-07

**Authors:** Timothy S. Horseman, Keith S. K. Fong, Jamie L. Dombach, Edwin Kamau, Alan P. Gehrich

**Affiliations:** 1 United States Army Institute of Surgical Research, Joint Base San Antonio–Fort Sam Houston, TX, United States; 2 Department of Clinical Investigation, Tripler Army Medical Center, Honolulu, HI, United States; 3 Department of Pathology, Tripler Army Medical Center, Honolulu, HI, United States; 4 Department of Obstetrics and Gynecology, Division of Urogynecology, Tripler Army Medical Center, Honolulu, HI, United States

**Keywords:** drug resistance, dysbiosis, *Enterobacteriaceae*/pathogenicity, *Lactobacillus*/physiology, microbial/genetics, postmenopausal, rectum/microbiology, urinary tract infections/microbiology

## Abstract

**Introduction:**

Recurrent urinary tract infections (rUTIs) in postmenopausal (PM) women pose a significant clinical challenge, complicated by rising antibiotic resistance among uropathogens. The vaginal microbiota in this population remains underexplored. We aim to characterize vaginal flora of PM women with and without a history of rUTIs, and to evaluate relationships to demographic variables, clinical characteristics, and rectal pathogen colonization.

**Methods:**

We conducted a cross-sectional study of 62 PM women (*n* = 31 rUTI, (*n* = 31 control). Vaginal swabs were analyzed using 16S rRNA and a next-generation sequencing tool designed to identify UTI pathogens and antibiotic resistance (AMR) markers. Rectal swabs were cultured to identify uropathogens and their phenotypic resistance. These were integrated with subject demographic and historical clinical data.

**Results:**

Compared to controls, rUTI vaginal microbiota exhibited a marked depletion in *Lactobacillus crispatus* and *L. iners*, species commonly associated with vaginal health, alongside an enrichment of *L. gasseri* and *L. jensenii*. The rUTI cohort also had a greater burden of AMR markers (*p* = 0.0003). Notably, Gram-negative uropathogens in the rUTI group frequently carried multidrug resistance genes, at rates nearly three times higher than controls. The rUTI cohort was further characterized by enrichment of Gram-negative uropathogens in the vagina. These alterations were more pronounced with increasing years in menopause.

**Discussion:**

The rectum emerged as a key reservoir, with notable concordance of organisms across rectal and urogenital sites. Our findings indicate that rUTIs in postmenopausal women are associated with a dysbiotic vaginal microbiome that is closely linked to a rectal reservoir of multidrug-resistant uropathogens.

## Introduction

The incidence of urinary tract infections (UTIs) in postmenopausal women is approximately 7% per year among community-dwelling women aged 55–75 based on prospective cohort data with microbiologically confirmed symptomatic cystitis (Jackson et al., 2004). Additionally, cross-sectional data indicate that the annual incidence among women older than 50 years is about 9% (Hu et al., 2004). The UTI recurrence risk in postmenopausal (PM) women is between 20 and 50% within 6–12 months after an initial episode (Jung and Brubaker, 2019). Further, complicating the management of UTIs in PM women are increasing antibiotic resistance rates. In this population, resistance to ampicillin exceeds 40%, while resistance to ciprofloxacin surpasses 25%. The overall resistance to commonly used antimicrobials including trimethoprim-sulfamethoxazole is higher in PM women compared to premenopausal women (Miotla et al., 2017). These factors increase the urgency to find new methods to treat and prevent UTIs in this population.

The pathogenesis of UTIs in women is characterized by the ascension of uropathogenic bacteria from the periurethral area into the bladder leading to cystitis. The female urethra’s short length and proximity to the anus, the reservoir for uropathogens, facilitate this process. Estrogen deficiency in PM women leads to atrophic changes in the vaginal and urethral mucosa allowing for increased colonization of uropathogens thereby increasing the risk of UTI. In addition, PM women more commonly suffer with aggravating factors such as prolapse, fecal and urinary incontinence, diabetes, incomplete bladder emptying, and poor hygiene which further increase their risk (Brubaker et al., 2018). Estrogen has a profound impact on the vaginal microbiome which includes increased thickness, ruggation, and secretions of the mucosa allowing for a predominance of *Lactobacillus* species which has been shown to inhibit the growth of uropathogens (Neugent et al., 2022; Leccese Terraf et al., 2017; de Llano et al., 2017; Johnson et al., 2022; Sekito et al., 2023). Vaginal estrogen supplementation in PM women has been shown to be an effective method of prophylaxis with a 50% reduction among patients with recurrent (r)UTI (Chen et al., 2021). The question remains why rUTIs affect only a subset of PM women. We seek to further elucidate the microbiologic factors that predispose PM women to lower urinary tract invasion by uropathogens, as well as sociodemographic, behavioral, and medical factors that may contribute to this vulnerability. Specifically, our study seeks to characterize the relationship between rectal pathogen colonization, vaginal microbiota and rUTIs. Insights into this relationship should improve strategies for the prevention and treatment of this common and debilitating condition.

## Methods

### Study participants and data collection

PM women were recruited from the Urology and Gynecology clinics at Tripler Army Medical Center (TAMC), Honolulu, HI and classified into one of two groups, recurrent urinary tract infections (rUTIs) or controls. Written informed consent was obtained from each patient with an interview completed at the time of enrollment to include past medical history, demographic information, and social history (Supplementary Figure 1).

Control participants were defined as having no menstrual periods for at least 12 months or having an elevated follicle-stimulating hormone level above 25 IU/L in cases where menstruation status could not be determined due to hysterectomy or endometrial ablation. The rUTI group included women with a history of at least three community acquired UTIs in the past year or two in the past 6 months, based on documented urine culture and antibiotic prescriptions. Controls had no more than one UTI in the preceding year. All subjects were required to have a minimum vaginal depth of 5 cm. Exclusion criteria included prolapse at or beyond hymen, post-void residual urine volume greater than 200 mL, use of self-catheterization, known immunodeficiencies, antibiotic or steroid use within the past 30 days and a history of anti-estrogen therapy or chemotherapy within the last year. Women were also excluded if they had a current diagnosis of bacterial vaginosis or a yeast infection.

Vaginal and rectal swab samples were collected from eligible, consented patients. Vaginal swab collections were taken from the mid-vaginal wall 3 cm interior to the introitus, avoiding contact with and possible contamination by bacteria from the introitus, or other vaginal sites. Vaginal swabs were preserved in a DNA/RNA Shield transport media (Zymo Research Corp., Irvine, CA, USA) and stored at 4 °C for no more than a week until extraction. Collection of paired rectal swabs were performed by carefully inserting both swab tips approximately 1 cm beyond the anal sphincter and rotating gently. Rectal swabs were stored at 4 °C for no more than 48 h before culture inoculation. All samples were de-identified according to the approved protocol.

### Rectal culture

Rectal swabs were cultured onto the following agar media: trypticase soy agar with 5% sheep blood (BD Life Sciences, Franklin Lakes, NJ, USA), chromogenic MacConkey (MAC, BD Life Sciences), HardyCHROM ESBL (Hardy Diagnostics, Santa Maria, CA, USA), CHROMID CARBA Smart (bioMérieux, Durham, NC, USA), and Spectra VRE (Remel, Lenexa, KS, USA). Media was incubated per the manufacturers’ recommendations. Bacterial growth was examined following incubation at 24 and 48 h. Colonies with distinct morphologies were subsequently isolated with identifications and antibiotic susceptibility testing performed using the VITEK 2 system (bioMérieux). Isolated colonies were then cryopreserved using MicroBank ceramic beads (Pro-Lab Diagnostics, Round Rock, TX, USA) and stored at −80 °C.

### Sequencing and bioinformatic analysis

Genomic DNA was purified from vaginal swabs using the ZymoBIOMICS DNA Miniprep Kit (Zymo Research) according to manufacturer instructions. Library preparation for 16S rRNA sequencing was performed by amplifying the V3–V4 regions following the “Illumina 16S Metagenomic Sequencing Library Preparation” protocol. DNA and libraries were quantified with the Qubit dsDNA HS Assay (Invitrogen Corp., Waltham, MA, USA) on a Qubit 4 (ThermoFisher, Waltham, MA, USA). Paired end-sequencing was performed on a MiSeq using a v3 × 600 cycle kit (Illumina, San Diego, CA, USA). Bioinformatic analysis for 16S data on FASTQ files was conducted using Quantitative Insights Into Microbial Ecology (QIIME2, version 2024.5). Initial processing, including demultiplexing and denoising, was performed with DADA2 with trimming and truncation parameters to maximize read quality and feature recovery (median quality score of >25). A phylogenetic tree was generated via SEPP fragment insertion. Taxonomic assignment of ASVs and classification into Community State Types (CST) (Waetjen et al., 2023) was performed using the Greengenes, Silva, RDP (GSR) curated database and classifier,^1^ which required processing via QIIME2-2023.2 due to compatibility constraints. Additionally, representative ASV sequences were queried in NCBI using BLASTn to obtain sequence similarities of unclassified taxa. This is treated as a supplement to GSR classification. ANCOM-BC was performed for taxonomic differential abundance with the threshold of significance set at *p* < 0.05 and LFC > 2. Sequences were deposited in the NCBI Sequence Read Archive.

Additionally, vaginal swab DNA was profiled using the Urinary Pathogen ID/AMR Panel (UPIP, Illumina) to identify antibiotic resistance genes among uropathogens in our cohorts. Library prep was performed following manufacturer protocols. Amplicons were pooled and sequenced 1 × 100 bp on the Illumina NextSeq1000 platform using a P1 × 100 cycle kit. Run data was uploaded to BaseSpace and the Explify UPIP Data Analysis App was used to identify microorganisms and antimicrobial resistance genes.

### Statistics

Statistical analysis and figure generation were performed using QIIME2, GraphPad Prism 10.3 (San Diego, CA, USA), IBM SPSS Version 29.0.1.1 (IBM, Armonk, NY, USA) and Microsoft Excel. Mann–Whitney test was used for comparisons such as control versus rUTI. Differences across ages were tested using a one-way analysis of variance (ANOVA) or Kruskal-Wallis as appropriate. Fisher’s Exact Test was used to evaluate differences in antibiotic resistance between groups. To evaluate associations between clinical metadata and bacterial genera, bivariate correlations were calculated using bivariate Spearman’s correlations were performed with the resulting *p*-values adjusted for multiple comparisons using the Benjamini-Hochberg procedure. A permutational analysis of variance (PERMANOVA) in QIIME2 was used to assess pairwise associations in beta diversity measures. ADONIS, a multivariate, distance-based metric was used to test if group centroids were different in a high-dimensional space. PCA plots were from QIIME2. Unless otherwise stated, values are represented as mean ± SEM. Statistical significance was set as *p* < 0.05.

## Results

### Subject demographics

A total of 62 post-menopausal women were enrolled, with equal numbers of participants with and without a history of rUTIs (*n* = 31 per group). Patient demographics and clinical characteristics are summarized in Table 1. The mean age of the control group was 67.1 ± 13.0 years compared with 69.5 ± 14.0 years in the rUTI group (*p* = 0.236), and the average number of years since the onset of menopause was also similar between groups (*p* = 0.88). Other key variables including body mass index, chronic hypertension, and hormone therapy did not differ significantly between groups. Although symptomatic urinary incontinence (48.4% vs. 35.5%) and fecal incontinence (12.9% v 3.2%) were more frequently reported among women with rUTIs, these differences were not statistically significant. Overall, the two groups were comparable with respect to demographics factors, co-morbidities, diet and lifestyle habits. Approximately two-thirds of participants in both groups were not on any type of estrogen therapy at the time of sample collection.

**Table 1:** Demographic, dietary and clinical variables in post-menopausal control and recurrent urinary tract infection subjects.

Factor	Control				Recurrent				*p*-value
	Mean	SD	Min	Max	Mean	SD	Min	Max	
Age	67.1	13.0	40.0	98.0	69.5	14.0	37.0	95.0	0.236
BMI	28.2	6.2	19.0	47.6	27.9	7.9	18.0	53.8	0.448
Weight (kg)	70.6	13.8	47.6	95.8	72.8	21.3	43.5	132.0	0.315
Height (in)	65.6	11.3	57.0	125.0	62.6	2.7	58.0	69.0	0.081
Number of children	2.3	1.7	0.0	8.0	2.2	1.3	0.0	5.0	0.408
Cesarean deliveries	0.2	0.7	0.0	3.0	0.2	0.6	0.0	3.0	0.358
Vaginal deliveries	2.0	1.8	0.0	8.0	2.1	1.3	0.0	5.0	0.466
Frequency of vaginal coitus per week	0.7	1.2	0.0	5.0	0.3	0.6	0.0	2.5	0.066
	***n***	**%**			***n***	**%**			
Years postmenapuasal									0.88
<10 years	10	32.3			9	29.0			
10–20 years	9	29.0			8	25.8			
>20 years	12	38.7			14	45.2			
Ethnicity									0.691
African American	2	6.5			1	3.2			
Asian	8	25.8			9	29.0			
Caucasian	14	45.2			14	45.2			
Laotian	1	3.2			1	3.2			
Mixed	5	16.1			3	9.7			
Pacific Islander	1	3.2			3	9.7			
Education									0.345
High school	10	32.3			5	16.1			
Some College	9	29.0			10	32.3			
College	9	29.0			9	29.0			
Postgraduate	3	9.7			7	22.6			
Smoking status									0.176
Non-smoker	29	93.5			24	77.4			
Former smoker	2	6.5			6	19.4			
Current smoker					1	3.2			
Daily fruit consumption									0.671
Low	13	41.9			16	51.6			
Med	14	45.2			11	35.5			
High	4	12.9			3	9.7			
Daily vegetable consumption									0.297
Low	13	41.9			16	51.6			
Med	13	41.9			7	22.6			
High	5	16.1			7	22.6			
Weekly meat consumption									0.233
Low	2	6.5			6	19.4			
Med	13	41.9			13	41.9			
High	16	51.6			11	35.5			
Exercise									0.804
Sedentary	9	29.0			7	22.6			
Moderate	15	48.4			17	54.8			
High	7	22.6			6	19.4			
Diabetes mellitus									0.74
No	25	80.6			26	83.9			
Yes	6	19.4			5	16.1			
Urinary incontinence									0.307
No	19	61.3			15	48.4			
Yes	12	38.7			16	51.6			
Symptomatic urinary incontinence									0.252
No	20	64.5			15	48.4			
Yes	11	35.5			15	48.4			
Symptomatic fecal incontinence									0.15
No	30	96.8			26	83.9			
Yes	1	3.2			4	12.9			
Wears protection									0.246
No	25	80.6			21	67.7			
Yes	6	19.4			10	32.3			
Stress urinary incontinence									1
No	21	67.7			21	67.7			
Yes	10	32.3			10	32.3			
Overactive bladder									0.096
No	18	58.1			12	38.7			
Yes	12	38.7			19	61.3			
Hysterectomy									0.375
No	19	61.3			15	48.4			
Yes	12	38.7			15	48.4			
Urogyn surgery history									0.058
None	27	87.1			19	61.3			
Anti-incontinence					5	16.1			
Prolapse	4	12.9			5	16.1			
Both					1	3.2			
Estrogen therapy at time of sample collection									0.114
None	22	71.0			21	67.7			
Vaginal	4	12.9			9	29.0			
Systemic	4	12.9							
Both	1	3.2			1	3.2			

SD = standard deviation; Min = minimum; Max = Maximum. Dietary consumption was self-reported.

The rUTI cohort had an average of 10.4 ± 7.4 UTIs in the 10 years preceding and up to 3 years following sample collection, all of which were treated with antibiotics (Supplementary Table 1). Women <10 years post-menopause had an average of 7.1 ± 3.5 UTIs, compared with 12.8 ± 8.9 UTIs in those 10–20 years post-menopause and 10.8 (±7.7) UTIs in those >20 years post-menopause (*p* = 0.218). The distribution of uropathogens differed across menopausal stages: *Escherichia coli* accounted for 77.2% of infections in the early postmenopausal group but declined to ~50% in the mid and late groups. Conversely, *Klebsiella pneumoniae* infections increased from 1.8% in early post-menopause to 34.8% in mid and 19.9% in late post-menopause (*p* < 0.001).

### Vaginal microbiota are altered in post-menopausal women with rUTIs

**Figure 1:**
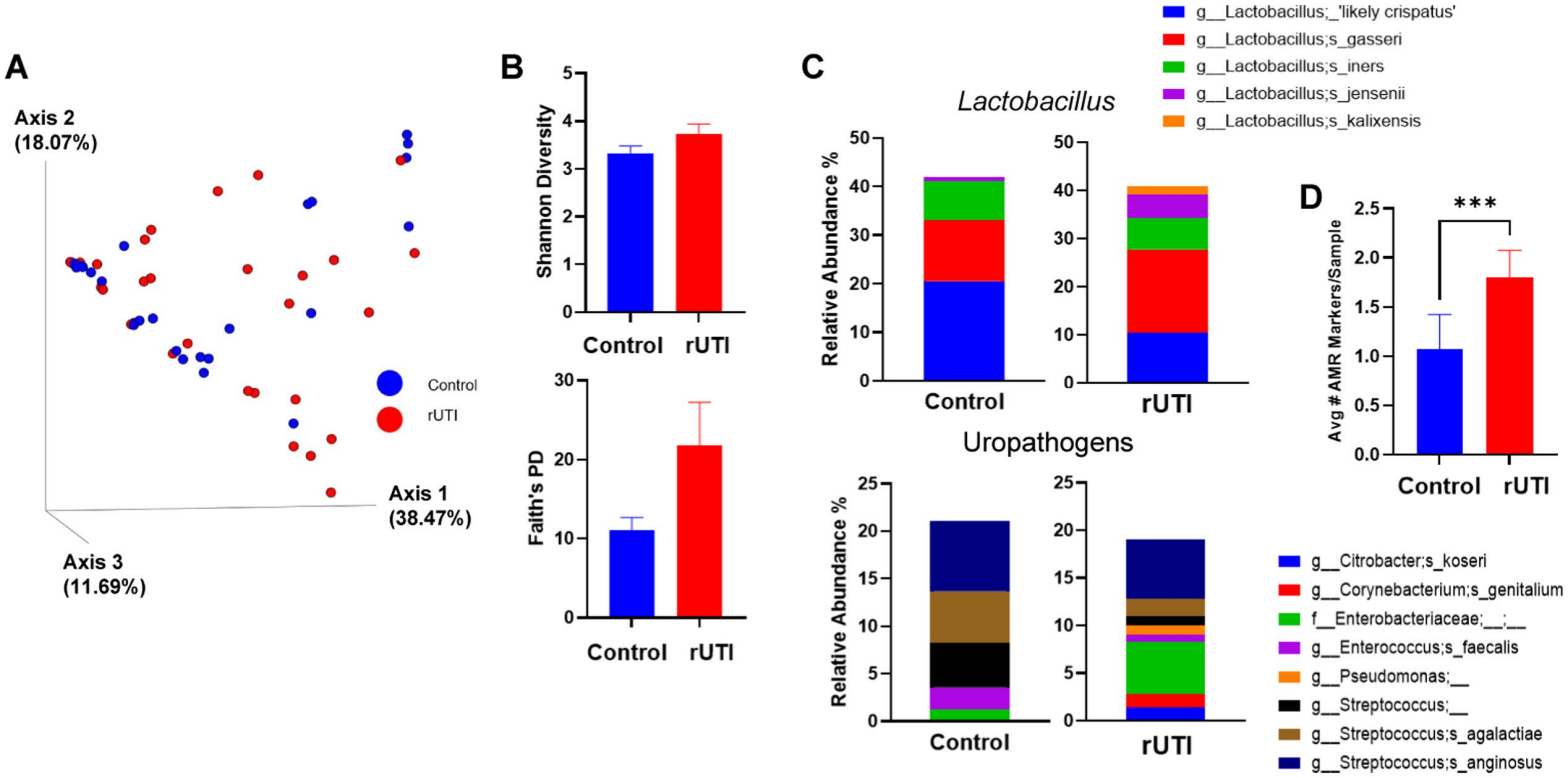
Post-menopausal vaginal microbiome composition and uropathogen abundance in recurrent urinary tract infections (rUTIs). **(A)** Principal coordinate analysis (PCoA) based on Generalized UniFrac evaluating phylogentic microbial community composition between control and rUTIs. Each axis of the plot explains data variance and each dot is an individual sample. **(B)** Alpha diversity (Shannon and Faith’s phylogenetic diversity) comparison demonstrating between post-menopausal groups. **(C)** Mean relative abundance of *Lactobacillus* species and common uropathogens showing in each group. **(D)** The bar graph displays the average number of antimicrobial resistance (AMR) markers detected per sample in healthy controls versus women with rUTIs. Bars represent mean ± SEM. Statistical significance was determined using a Mann–Whitney *U* test, *** *p* < 0.001. *n* = 31/group.

Analysis of the vaginal microbiome revealed no significant differences in overall microbial community structure or alpha diversity between controls and rUTI subjects (Bray-Curtis, *p* = 0.34; Generalized UniFrac distances, *p* = 0.15, Figure 1A; Shannon and Faith’s PD, *p* = 0.13–0.14, Figure 1B).

At the genus- and species-level, both groups exhibited similar mean relative abundances of *Lactobacillus* and uropathogens (Figure 1C). However, the rUTI group showed depletion of an unclassified species of *Lactobacillus* (based on BLAST - *L. crispatus*) and *L. iners*, with corresponding increases in *L. gasseri*, *L. jensenii*, and *L. kalixensis* (Supplementary Data 1). Although not significant, controls tended to have higher levels of Gram-positive uropathogens (e.g., *Streptococcus* and *Enterococcus*), whereas rUTI subjects exhibited enrichment of facultative anaerobes including *Prevotella* and members of the *Enterobacteriaceae*.

To further evaluate taxonomic patterns, we defined a “dominant” genus as one that comprised > 50% of relative abundance (Supplementary Figure 2–stacked barplots from every subject). A dominant genus was present in 90% of controls compared with 66.7% in the rUTI subjects, suggesting a shift toward higher community diversity in rUTIs. In both groups, *Lactobacillus* was the most frequent dominant genus (43.3% of controls and 50% of rUTI). *Streptococcus* was the second most frequent dominant genus among controls (20%), and a minority of individuals in both cohorts were dominated by *Bifidobacterium*, *Gardnerella* or other genera. Cervicovaginal community state typing (CST) further supported these findings: the high-diversity CST IV was the most prevalent in both groups (PM = 16; rUTI = 16), whereas CST V was observed exclusively in rUTI subjects (Supplementary Table 2).

Differential abundance testing with ANCOM-BC identified only one species with statistically significance differences between groups: *Corynebacterium coyleae* was significantly depleted in rUTI subjects (log fold change (LFC) = −1.1, *q* = 0.039). Two additional taxa showed notable but non-significant trends, with *Dialister microaerophilus* decreased in rUTIs (LFC = −1.53, *q* = 0.078) and *Enterobacteriaceae* enriched in the rUTI cohort (LFC = 2.32, *q* = 0.41).

To further characterize the differences between cohorts, antimicrobial resistance (AMR) genes were profiled using UPIP sequencing. The rUTI group had significantly higher levels of AMR markers compared to the control group (*p* = 0.0003, Figure 1D). Although AMR genes were detected in both groups, rUTI subjects were distinguished by Gram-negative uropathogens harboring multidrug resistance profiles: 58.6% of Gram-negatives from the rUTIs carried carbapenem and fluoroquinolone resistance genes, nearly three times the proportion observed in controls (20%; *p* = 0.0092). In contrast, AMR markers in controls were more commonly associated with Gram-positives organisms such as *Enterococcus* and *Streptococcus*, though these differences were not statistically significant (*p* = 0.74; Supplementary Data 2).

### Time post-menopause and rUTI status synergistically alter the vaginal microbiota

**Figure 2:**
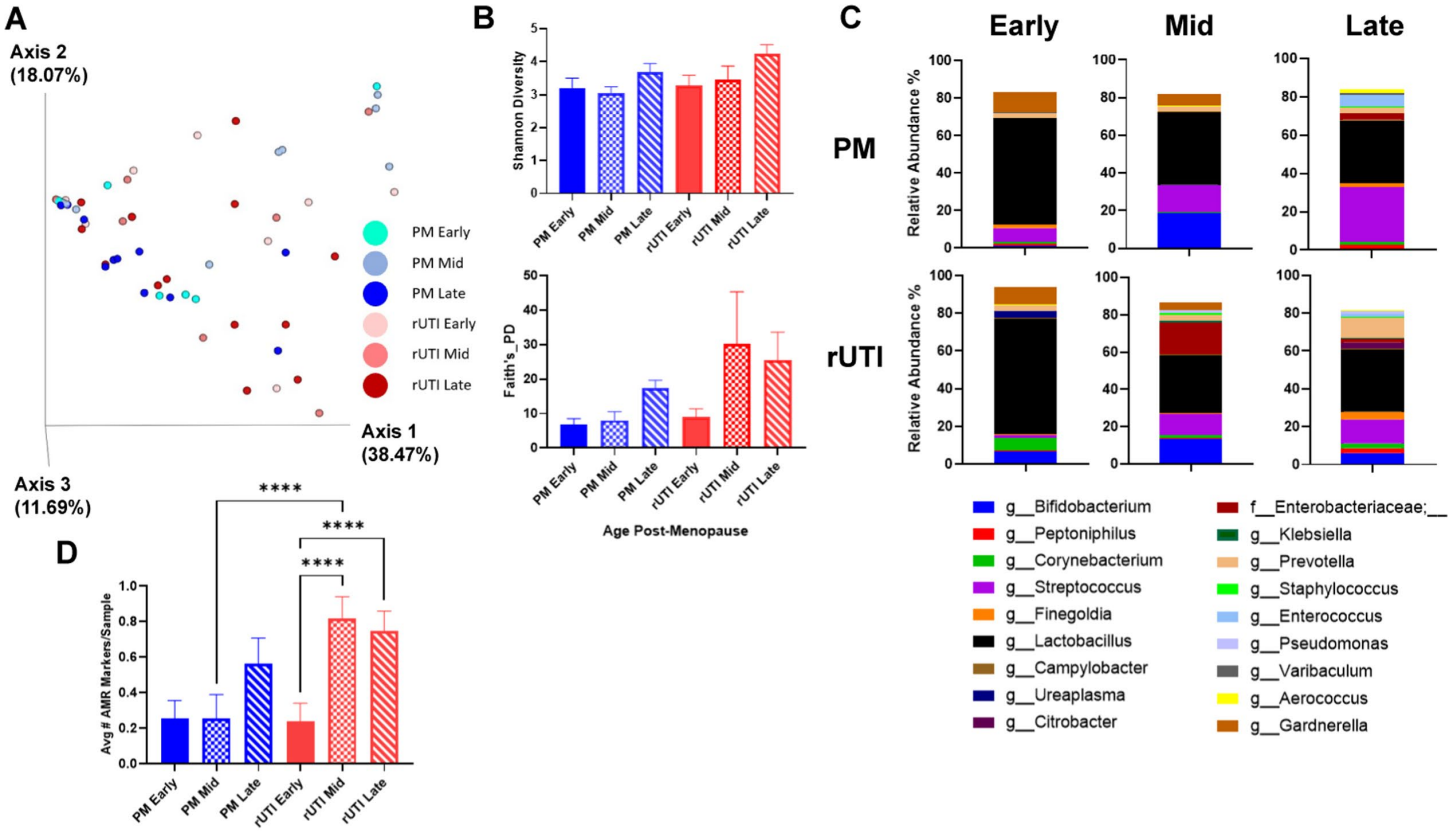
Impact of years since menopause on vaginal microbiome composition between controls and recurrent urinary tract infections (rUTIs). **(A)** Represents generalized UniFrac distances comparing microbial community stability across healthy post-menopausal controls (PM) and recurrent UTI (rUTI) age groups. Each dot represents a subject, and colors indicate different age groups within each cohort. **(B)** Shannon diversity and Faith’s phylogenetic diversity in control and rUTI samples across early, mid, and late stratified age groups. **(C)** Stacked barplots show dominant genera (>1% mean relative abundance) in each age group compared between post-menopausal cohorts. **(D)** The bar graph compares the mean number of antimicrobial resistance (AMR) markers identified per sample for both the PM control (blue) and rUTI (red) cohorts across three menopausal stages: early (solid), mid (checkerboard), and late (striped). PM (early, *n* = 9; mid, *n* = 10; late, *n* = 12) and RUTI (early, *n* = 9; mid, *n* = 8; late, *n* = 13). Bars represent mean ± SEM. ***p < 0.0001.

To assess the impact of aging on the vaginal microbiome, subjects were stratified into three postmenopausal stages: <10 years, 10–20 years, and >20 years since the onset of menopause. Although community diversity was modestly altered across age groups (Figures 2A,B), community structure shifted more substantially. The prevalence of single taxon dominance (>50%) remained high among controls (82–100%) but declined with increasing years post-menopause in the rUTI cohort, from 90% in early post-menopause to 46% in the late stage.

These age-related shifts were driven by changes in taxonomic composition in both groups. *Lactobacillus* remained the dominant genus across all stages, although its relative abundance declined with increasing time in menopause (Figure 2C). Species-level profiles differed between age groups and cohorts (Supplementary Figure 3). Among controls, *Lactobacillus* species were generally less diverse across age groups with unclassified *Lactobacillus* sp. (based on BLAST - *L. crispatus*) and *L. iners* dominating the early stage. While mid and late controls shifted toward *L. gasseri* and lower levels unclassified *Lactobacillus*. In contrast, rUTI subjects exhibited consistently high *L. gasseri* across stages, with *L. jensenii*, *L. iners*, and the unclassified *Lactobacillus* appearing only transiently. The CST distributions for each menopausal stage are shown in Supplementary Table 3.

Opportunistic taxa increased with time since menopause, particularly among rUTI subjects. Both cohorts had decreased *Gardnerella* and increased *Peptoniphilus*, *Streptococcus*, and *Enterococcus* in the mid and late stages. rUTI subjects demonstrated additional enrichment of *Enterobacteriaceae* (notably in mid stage), and *Finegoldia*, and *Prevotella* in the mid and late stages. Among women with low *Lactobacillus* (<50% relative abundance), controls were predominantly colonized by Gram-positive organisms like *Streptococcus* (41.1%), whereas rUTIs were more often dominated by Gram-negative genera, including *Enterobacteriaceae* and *Prevotella* (35.3%). Differential abundance analysis did not identify any taxa reaching statistical significance.

Age-related taxonomic transitions paralleled increases in AMR gene burden, with distinct trajectories between cohorts (Figure 2D; Supplementary Data 2). Gram-negative resistance to carbapenems and fluoroquinolones remained high and stable among rUTI subjects (53–63%), whereas controls showed a modest upward trend (11.1–30%; *p* = 0.66–0.85). For Gram-positives, both cohorts exhibited similar increases in aminoglycoside resistance from early to late post-menopause (controls: 22.2–41.7%; rUTIs: 11.1–38.5%; *p* = 0.61 and *p* = 0.39, respectively).

### Clinical variables help shape the vaginal microbiota in post-menopausal women

**Figure 3:**
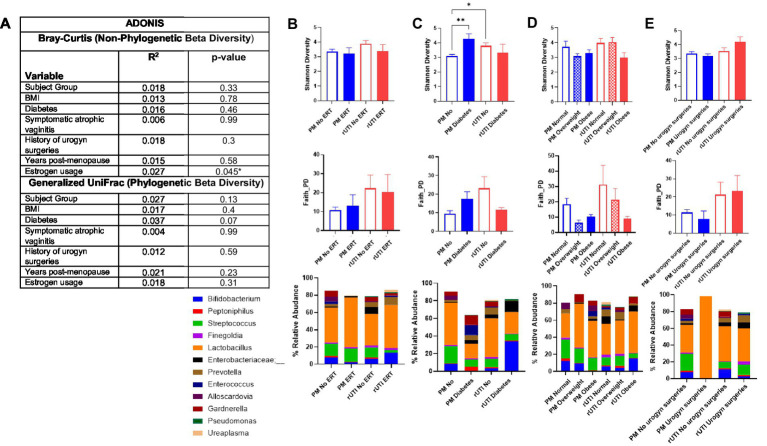
Vaginal microbiota and associated clinical characteristics of recurrent UTI. **(A)** ADONIS multivariate analysis of beta diversity metrics (Bray-Curtis and generalized UniFrac) and the influence of each demographic or clinical characteristic on microbial community composition alterations. **(B–E)** Along the top is a comparison of alpha diversity metrics (Shannon and Faith’s phylogenetic diversity) followed by barplots below of mean relative abundance of common genera between control and rUTI subject with and without a particular category. *p < 0.05, **<0.01. ERT = estrogen replacement therapy. Body mass index (BMI) stratified into three groups: Normal (<24.9), overweight (25–29.9), and obese (>30). Urogyn surgeries = History of urogynelogical surgeries. Sample sizes per group PM (No ERT, *n* = 25; ERT, *n* = 6; No diabetes, *n* = 25; Diabetes, *n* = 6; Normal, *n* = 9; Overweight, *n* = 11; Obese, *n* = 11; No urogyn surgeries, *n* = 27; urogyn surgeries, *n* = 4). rUTI (No ERT, *n* = 22; ERT, *n* = 9; No diabetes, *n* = 26; Diabetes, *n* = 5; Normal, *n* = 11 Overweight, *n* = 11; Obese, *n* = 9; No urogyn surgeries, *n* = 20; urogyn surgeries, *n* = 11).

Potential clinical and demographic contributors to rUTIs and reduced *Lactobacillus* abundance were evaluated (Supplementary Figure 4). Menopausal estrogen therapy at the time of sample collection was the only variable significantly associated with microbial community composition (Bray-Curtis, *p* = 0.045; Figure 3A). Although estrogen use did not significantly affect alpha diversity, it was associated with a healthier microbial profile in rUTI subjects, characterized by higher *Lactobacillus* relative abundance and lower levels of *Streptococcus* and *Enterobacteriaceae* – (Figure 3B). Within the rUTI cohort, estrogen use also correlated with higher *Lactobacillus* level (*p* = 0.083; Supplementary Figure 4A).

Type II diabetes mellitus was another factor influencing the vaginal microbiome. Among controls, diabetic women had significantly higher Shannon diversity than non-diabetic controls (*p* = 0.01; Figure 3C). Across both cohorts, diabetes was strongly associated with low *Lactobacillus* abundance: 29.4% of diabetic controls and 23.5% of diabetic rUTI subjects had low *Lactobacillus*, compared with only 7.7% of non-diabetic women in either group. A history of urogynecologic surgery was more common among rUTI subjects (35–38% vs. 12–15% of controls; *p* = 0.058). No significant associations were observed for BMI or other examined variables (Figures 3D,E).

### Association of vaginal uropathogen colonization with rectal reservoir and a dysbiotic microbiome

**Figure 4:**
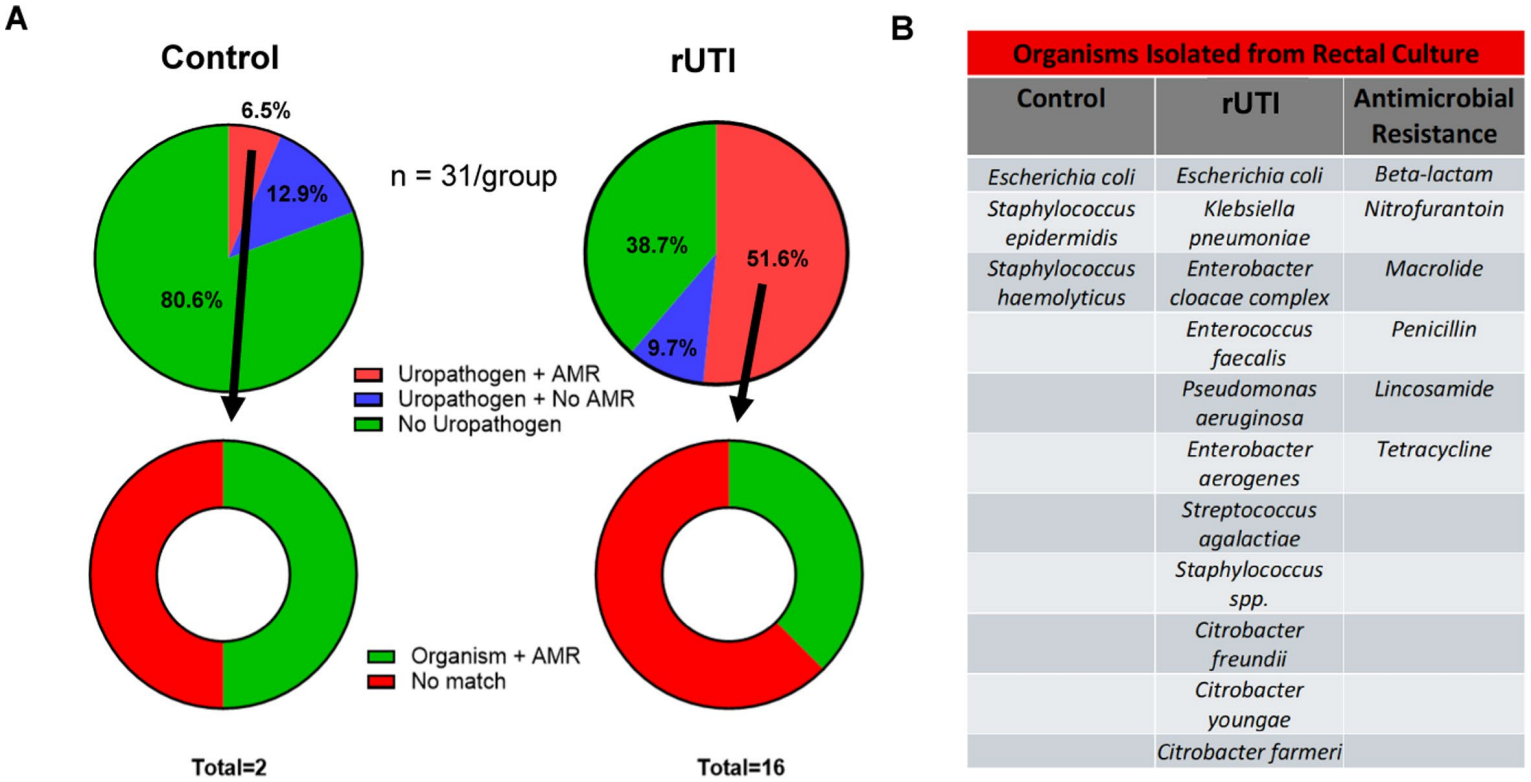
Concordance of organisms and antimicrobial resistance between rectal culture and vaginal swab UPIP sequencing. **(A)** The top pie charts represent the prevalence of rectal colonization with uropathogens and antimicrobial-resistance (AMR) between control (*n* = 31) and rUTI (*n* = 31) cohorts. The bottom donut figures show concordance of uropathogen and AMR matches from rectal cultures of each cohort and corresponding vaginal microbiota data from UPIP sequencing. **(B)** The table lists the specific organisms isolated from rectal cultures and the antimicrobial resistance classes identified.

Using UPIP sequencing, we identified genetic resistance determinants of vaginal uropathogens (Supplementary Data 2). To assess a potential reservoir for these organisms, we performed rectal cultures from the same participants and compared rectal isolate with vaginal UPIP sequencing results and historical urine cultures. Rectal colonization with phenotypically resistant uropathogens was significantly more common in the rUTI cohort: 38.7% of rUTI subjects harbored an antibiotic-resistant organism compared to only 6.5% of controls (*p* = 0.0058; Figure 4A). In rUTI subjects, the most frequently isolated organisms with antibiotic resistance organisms included *E. coli*, *K. pneumoniae*, *Pseudomonas aeruginosa*, and *Enterococcus faecalis*, with resistance most often observed against beta-lactams, tetracycline, and macrolides (Figure 4B). Notably, 37.5% of rectal isolates in rUTI cohort showed phenotypic resistance profiles that matched both the organism and AMR genotype detected in the vagina via UPIP sequencing.

We observed substantial concordance across rectal cultures, vaginal UPIP sequencing, and historical urine culture results. Among the 29 rUTI subjects with prior *E. coli* UTIs, 78.6% were rectally colonized with *E. coli*, while 39.3% showed vaginal carriage. The second most common historical uropathogen, *K. pneumoniae*, was documented in 33.3% of rUTI subjects, detected in 32.1% of rectal cultures, and identified in 14.3% of vaginal samples. Across datasets, five organisms demonstrated complete concordance across all three sources - rectal, vaginal, and urine. Additional overlaps included four organisms shared only between rectal and urine cultures, one shared between rectal and vaginal samples, and two between vaginal samples and urine cultures.

The connection between the rectal reservoir and UTIs was further supported by comparing phenotypic resistance profiles from historical urine cultures and rectal isolates with corresponding genotypic resistance markers identified in vaginal UPIP sequencing. When matching both organism and AMR profile, we found six subjects with concordance across rectal, vaginal, and urine sample sites. An additional six subjects showed concordance exclusively between rectal and urine cultures, one between rectal and vaginal samples, and three between vaginal and urine samples.

Two cases illustrate the strong concordance across rectal, vaginal and urinary sites. Subject rUTI1 had a history of *K. pneumoniae* UTIs resistant to ampicillin and nitrofurantoin. The rectal culture from this patient grew *K. pneumoniae* with the same phenotypic resistance profile, and vaginal UPIP sequencing identified *K. pneumoniae* carrying the ACT-52 and MIR-3 beta-lactamase genes, which confer ampicillin resistance. Subject rUTI29 provides another clear example: vaginal sequencing detected *E. coli* strain harboring the *ampH* beta-lactamase gene along with fluoroquinolone-resistance mutations in *gyrA* and *parC*, matching the beta-lactams and fluoroquinolones resistance phenotypes observed in both the rectal and historical urine isolates. These cases highlight the rectum as a reservoir seeding the vagina and urinary tract with the same resistant strains.

## Discussion

The findings of our study largely align with and reinforce the established literature regarding the contribution of the vaginal microbiome on the pathophysiology of rUTIs in postmenopausal women. We demonstrate that rUTI status was associated with: (i) a species-level shift within *Lactobacillus*—depletion of *L. crispatus* and *L. iners* alongside enrichment of *L. gasseri*, *L. jensenii*, and late emergence of *L. kalixensis*; (ii) greater alpha diversity and reduced single-taxon dominance with increasing time since menopause; (iii) enrichment of facultative anaerobes and *Enterobacteriaceae*; and (iv) a higher burden of antimicrobial resistance (AMR) markers in the vaginal microbiome and on rectal culture in postmenopausal women with rUTI. Importantly, our study adds strong evidence documenting the transfer of uropathogenic organisms along the gut/vagina/bladder axis by linking vaginal pathogens to a rectal reservoir.

*Lactobacillus* has been identified as the principal organism in the vagina associated with protection against UTIs (Stapleton, 2016). Our findings challenge the paradigm that *Lactobacillus* abundance at the genus level is the sole determinant of vaginal health in PM women, our findings indicate that species-level differences are more biologically consequential. *L. crispatus* predominance in the vaginal flora has been linked to lower vaginal pH, higher levels of lactic acid and bacteriocin production, and resistance to colonization by uropathogens (Stapleton, 2016), (He et al., 2020; Argentini et al., 2022). This aligns with functional studies on CSTs showing that *L. crispatus* predominant CST I are superior to CST II (*L. gasseri*) and CST V (*L. jensenii*) in adhesion prevention, acidification, and pathogen inhibition (He et al., 2020; Argentini et al., 2022; de Oliveira et al., 2021). In a study of postmenopausal women undergoing urogynecologic surgery, women who had decreased *L. iners*, were more likely to develop UTIs postoperatively (Thomas-White et al., 2018). Our findings reinforce that genus-level metrics can obscure functionally divergent species compositions.

The limited success of *Lactobacillus* recolonization therapies for rUTI may be explained by these species-level dynamics and the difficulty of displacing established uropathogens. Our data hint that a healthy vaginal microbiota may depend on a consortium of protective bacteria with the combined depletion of *L. crispatus*, *L. iners*, and *C. coyleae*, creating a niche for uropathogen invasion (Dalby et al., 2025). Future studies should rely on whole genome sequencing to define quantitative abundance thresholds of protective *Lactobacillus* species and strains required for colonization resistance.

Simple diversity metrics have not been consistently associated with rUTI status (Sekito et al., 2023; Neugent et al., 2022; Park et al., 2023).

We showed higher alpha diversity, a trend toward separation in beta diversity, and reduced single-taxon dominance among our cohort of rUTIs. As such, diversity measures are likely not adequate to determine predisposition to rUTIs. We observed alterations in taxonomic community composition with respect to time since menopause with the loss of *Lactobacillus* dominance and species turnover more pronounced in rUTI subjects, consistent with a state of microbiome instability/dysbiosis (Park et al., 2023; Neugent et al., 2022; Hugenholtz et al., 2022). Our findings suggest that taxonomic changes rather than diversity are a more appropriate measure of rUTI risk. Interestingly, this microbial instability may in part arise from antibiotic use, creating a problematic cycle with ever decreasing protective bacteria and increasing uropathogens in the gut, vaginal and bladder microbiome predisposing postmenopausal women to rUTIs (Bhuiya et al., 2025; Dutta et al., 2024; Siddiqui and Bradley, 2025).

Local estrogen therapy has a proven track record in preventing UTIs in postmenopausal women. Meta-analyses and randomized trials indicate vaginal estrogen reduces rUTI recurrence and lowers vaginal pH (Chen et al., 2021; Ferrante et al., 2021; Ackerman et al., 2026; Brubaker et al., 2018). This is supported by our analysis. Postmenopausal women on vaginal estrogen show increased *Lactobacillus* and those with rUTIs had reduced uropathogens within the vaginal flora as compared to those without vaginal estrogen. These observations align with urogenital microbiome studies demonstrating estrogen-associated enrichment of *Lactobacilli* and *Bifidobacteria* in PM women (Neugent et al., 2022; Srinivasan et al., 2022). The effect of local estrogen therapy on UTI recurrence is likely not limited to promotion of *Lactobacillus* but also the reduction in vaginal uropathogen colonization. This has not been rigorously tested in clinical trials but the 50–75% reduction in UTI recurrence with local estrogen therapy provides strong indirect support that vaginal estrogen reduces uropathogenic colonization (Ackerman et al., 2026).

A critical finding of our study is the high rectal colonization with antibiotic-resistant uropathogens among rUTI subjects. Paired rectal cultures and vaginal sequencing revealed high concordance of organism and AMR profiles across the rectal, vaginal, and urine sites, which strongly supports a gut-to-vagina-to-bladder reservoir pathway for recurrence (Schembri et al., 2022; Worby et al., 2022; Dutta et al., 2024). These findings align with previous multi-omics longitudinal studies demonstrating gut dysbiosis (loss of butyrate producers), persistent gut uropathogenic *E. coli* reservoirs post-antibiotic treatment, and systemic inflammatory signatures in women with rUTI (Magruder et al., 2019; Brannon et al., 2020). The clinical implications are significant: AMR genes accumulate in PM urogenital microbiomes with a history of rUTI, even during remission. Molecular-based assays can rapidly detect and map these AMR markers to pathogens, providing a valuable diagnostic tool for targeted antibiotic stewardship (Gabor Fidler et al., 2025; Neugent et al., 2022).

We propose that depletion of *L. crispatus* and possibly *L. iners* diminishes lactic acid/bacteriocin output and mucosal barrier function, raising pH and reducing colonization resistance, thereby permitting expansion of *Enterobacteriaceae* and anaerobes (e.g., *Prevotella*). In rUTI subjects, persistence of less protective *L. gasseri* and increasing community diversity may be insufficient to suppress uropathogen colonization, especially when gut reservoirs continuously reseed the vagina and bladder (Neugent et al., 2020; He et al., 2020; Argentini et al., 2022; Brannon et al., 2020; Schembri et al., 2022).

Effective management of rUTI in postmenopausal women requires a multifaceted approach informed by microbiome insights. First, species-level diagnostics should replace genus-level assessments, moving beyond “Is *Lactobacillus* present?” to “Which species?”— with emphasis on restoring protective species such as *L. crispatus* (Neugent et al., 2020; He et al., 2020; Argentini et al., 2022; de Oliveira et al., 2021; Neugent et al., 2022). Second, reservoir surveillance is critical; paired rectal–vaginal–urine sampling combined with AMR genotyping can identify re-seeding cycles and guide therapy (Schembri et al., 2022; Neugent et al., 2022). Third, microbiome-targeted strategies should include vaginal estrogen for postmenopausal women, which has demonstrated efficacy in reducing rUTI recurrence, (Ferrante et al., 2021; Chen et al., 2021) alongside probiotic approaches (including *L. crispatus*-containing preparations) supported by clinical evidence, though strain validation and rigorous trial design remain essential (Stapleton, 2016; Schembri et al., 2022; Gupta et al., 2024). Finally, stewardship and AMR-informed therapy must be prioritized: incorporating AMR profiling to tailor antibiotic use and integrating non-antibiotic adjuncts, as guidelines increasingly emphasize minimizing unnecessary antibiotic exposure in rUTI care (Brubaker et al., 2018; Ackerman et al., 2026; Naji et al., 2024; Lynch et al., 2025).

The strengths of the study include multi-modal profiling (16S and UPIP sequencing, rectal culture with phenotypic antimicrobial susceptibility testing) and cross-site concordance anchoring ecological interpretation. This study has several limitations to consider when interpreting data. First, the cross-sectional design limits causal inference between vaginal microbial dysbiosis, AMR burden, and rUTIs. Although we attempted to limit the effect of antibiotic treatment on the vaginal microbiome with obtaining samples more than 30 days from most recent antibiotic treatment, studies have shown that repeated courses of antibiotics disrupt the vaginal microbiome and predispose women to the less protective *L. gasseri* dominated vaginal microbiome (Sekito et al., 2023; Bhuiya et al., 2025), Additionally, the single collection time point makes it difficult to determine whether the observed microbial alterations predispose women to rUTIs or are a consequence of repeated infections and antibiotic use. Longitudinal studies evaluating the urogenital microbial profiles before, during, and following UTIs are required to elucidate these relationships (Dalby et al., 2025). Subgroup sample sizes (some covariate analyses underpowered), and species-level constraints of 16S sequencing are also limitations of our study (Adeolu et al., 2016; Janda and Abbott, 2021). Whole genome sequencing is needed to provide strain-level taxonomic and functional data to address the potential variation of antimicrobial activity among *Lactobacillus* species and provide actionable therapeutic targets.

In summary, rUTI in postmenopausal women is characterized less by genus-level shifts and more by species-level loss of protective lactobacilli—notably *L. crispatus* (and, possibly, *L. iners*) —together with greater AMR burden and organism/AMR concordance across rectal, vaginal, and urinary sites, consistent with a gut-to-vagina-to-bladder reservoir pathway. These findings help reconcile why total *Lactobacillus* abundance may appear “normal” in some rUTI cases, yet colonization resistance is diminished and facultative anaerobes/*Enterobacterales* gain a foothold. Clinically, they support species-resolved diagnostics, AMR-informed stewardship, and adjuncts that restore protective species (e.g., *L. crispatus*) in concert with rectal reservoir surveillance and estrogen therapy where appropriate. Future longitudinal and interventional studies—ideally integrating shotgun metagenomics and strain-level tracking—are needed to confirm directionality and to test targeted strategies that stabilize the postmenopausal vaginal niche and interrupt reservoir-driven recurrence.

## Data Availability

The original contributions presented in the study are included in the article/Supplementary material, further inquiries can be directed to the corresponding author.

## Footnotes

1 https://manichanh.vhir.org/gsrdb/,version V3–V4.

## Author disclaimer

The views expressed in this study are those of the authors and do not necessarily reflect the official policy or position of the Defense Health Agency, Department of Defense, nor the U.S. Government. This work was prepared as part of official duties. Title 17, U.S.C., Section 105 provides that copyright protection under this title is not available for any work of the U.S. Government. Title 17, U.S.C., Section 101 defines a U.S. Government work as a work prepared by a military service member or employee of the U.S. Government as part of that person's official duties.

## Supplementary material

SUPPLEMENTARY FIGURE 1 Patient questionnaire.

SUPPLEMENTARY FIGURE 2 Stacked bar chart illustrates the mean relative abundance of different bacterial genera for each individual sample divided by post-menopausal group. Each vertical bar represents a single sample, with subject IDs labeled on the *x*-axis (P = Postmenopausal Control, R = rUTI). The y-axis indicates the relative frequency of each taxon and different colors correspond to distinct bacterial genera.

SUPPLEMENTARY FIGURE 3 The stacked barplots show the distribution of Lactobacillus species among age groups post-menopause and between cohorts (controls (PM) versus rUTIs).

SUPPLEMENTARY FIGURE 4 Bars represent the percentage of subjects within each of four pre-defined cohorts who reported a specific clinical characteristic (*x*-axis). Postmenopausal controls with low Lactobacillus (<50% relative abundance; PM Low Lactobacillus; *n* = 17), postmenopausal controls with high Lactobacillus (>50%; PM High Lactobacillus; *n* = 14), rUTI subjects with low Lactobacillus (rUTI Low Lactobacillus; *n* = 17), and rUTI subjects with high Lactobacillus (rUTI High Lactobacillus; *n* = 14). The *y*-axis displays percent of subjects within each of those specific cohorts who have the characteristic listed on the *x*-axis. ‘Atrophic vaginitis’ includes only symptomatic cases. ERT, Estrogen Replacement Therapy.

SUPPLEMENTARY TABLE 1 UTI history.

SUPPLEMENTARY TABLE 2 Community State Types among Postmenopausal Women with and without recurrent Urinary Tract Infections. This table presents the number of individuals able to be categorized into five different Community State Types (labeled I-V). It compares the distribution of these types between postmenopausal women with recurrent urinary tract infections (Recurrent UTI group) and a Postmenopausal Control group. CST – Community State Type- a classification of the vaginal microbiome; UTI – urinary tract infection. #Total number of CSTs for rUTI were 27. Vaginal microbiomes from four subjects could not be categorized into one of the CSTs and are therefore not reported.

SUPPLEMENTARY TABLE 3 Community State Types among Postmenopausal Women with and without recurrent urinary tract infections separated by time in menopause. Distribution of five distinct Community State Types (CSTs I-V) comparing the Control group (postmenopausal women without recurrent urinary tract infections) and the rUTI group (postmenopausal women with recurrent urinary tract infections). Within each group, participants are further stratified by their time in menopause: Early, Mid, and Late stages. The bottom table provides an aggregated view, showing the Combined counts for both Control and rUTI participants across all categories. Each cell value represents the number of individuals fitting the specified CST and subgroup. Abbreviations: CST: Community State Type, a classification of the vaginal microbiome. rUTI: Recurrent Urinary Tract Infection. $- Group differences were not assessed due to small sample sizes # Due to small sample sizes, to assess the association between years menopause and CST, we dichotomized CST groups as I-III and IV/V, and menopausal years as early and mid/late. This showed a trend toward CST IV/V in mid/late menopause compared to early (65.9% vs. 41.2%, *p*=0.142).

SUPPLEMENTARY DATA 1 Summary of organisms detected from vaginal swabs using UPIP sequencing. The table details subject ID, mean abundance and organisms identified with genotypic antimicrobial resistance markers and corresponding drug classes.

SUPPLEMENTARY DATA 2 Amplicon Sequence Variants (ASVs) and taxonomic assignment for each ASV using the Basic Local Alignment Search Tool (BLAST) across all samples.
