# Supplementary material for: Vaginal microbiome dysbiosis and a rectal reservoir of uropathogens characterize postmenopausal women with recurrent urinary tract infections: a cross-sectional study
Source: Front Microbiol. 2026 Apr 7;17:1812000. doi: 10.3389/fmicb.2026.1812000 (PMC13096829; doi:10.3389/fmicb.2026.1812000)
Supplement: SUPPLEMENTARY FIGURE 1 — Patient questionnaire. [file Presentation_1.pdf]

<u>Patient Interview Questionnaire</u>

Ethnicity:

- Caucasian
- African-American
- Asian
- Pacific Islander
- Hispanic
- Other: ____

Smoking Status:

- Current smoker
- Ex-smoker---how long did you smoke?
- Non-smoker

Education Level:

- Some high school
- High school
- Some college
- Bachelor’s degree
- Graduate/professional degree
- Other:____

Diet:

- Vegan
- Vegetarian
- Omnivore

What is your daily consumption of fruit?

Low = 0-2 portions/day

Moderate = 3 portions/day

High = ≥ 4 portions/day

What is your daily consumption of vegetables?

Low = 0-2 portions/day

Moderate = 3 portions/day

High = ≥ 4 portions/day

What is your weekly consumption of meat and typically what type?

Low = never to once/week

Moderate = 2-4 times/week

High = 5-7 times/week

White meat (chicken, turkey, etc….)

Red meat (beef, pork, etc….)

Fish

How often do you eat out to include fast food?

Low = never to once/week

Moderate = 2-4 times/week

High = 5-7 times/week

Comments

Exercise:

Please rate your physical activity based on the following scale from 1-10:

Sedentary (1-4): Physically active less than 30 minutes/day for 5 days a week

Moderately-active (5-7): At least 30 minutes of exercise/day for 5 days a week

Highly Active (8-10): Vigorous-intensity activities for a minimum of 30 minutes three days per week

Comments

Number of Children? Method of delivery for each?

Sexual Preference?

- Homosexual
- Heterosexual
- Bisexual

- Other: ____

Frequency of vaginal intercourse? On average, how many times per week in the last year?

Frequency of condom use?

- Never
- Sometimes
- Always

Do you have a history of STIs?

Are you on hormone/estrogen replacement therapy? If so, dosage and duration? (confirm with medical record)

Do you have a family or personal history of UTIs?

Antibiotic/steroid/chemotherapy history?

Co-morbidities? (Example: Diabetes, etc…)
