# Supplementary figures and images for: Vaginal microbiome dysbiosis and a rectal reservoir of uropathogens characterize postmenopausal women with recurrent urinary tract infections: a cross-sectional study
Source: Front Microbiol. 2026 Apr 7;17:1812000. doi: 10.3389/fmicb.2026.1812000 (PMC13096829; doi:10.3389/fmicb.2026.1812000)

## Slide 1
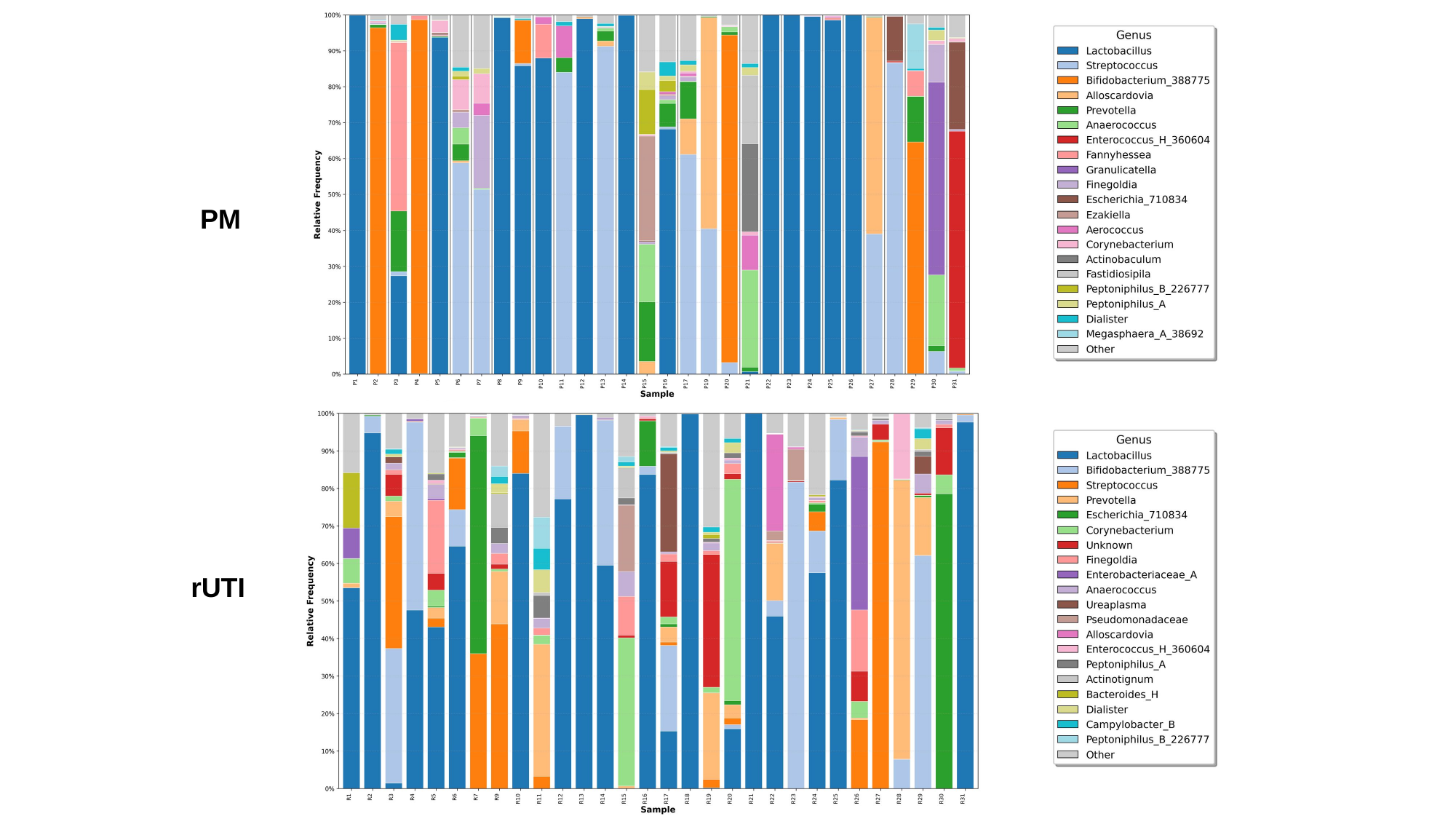

PM
rUTI

Supplement: SUPPLEMENTARY FIGURE 2 — Stacked bar chart illustrates the mean relative abundance of different bacterial genera for each individual sample divided by post-menopausal group. Each vertical bar represents a single sample, with subject IDs labeled on the x-axis (P = Postmenopausal Control, R = rUTI). The y-axis indicates the relative frequency of each taxon and different colors correspond to distinct bacterial genera. [file Presentation_2.pptx]

## Slide 1
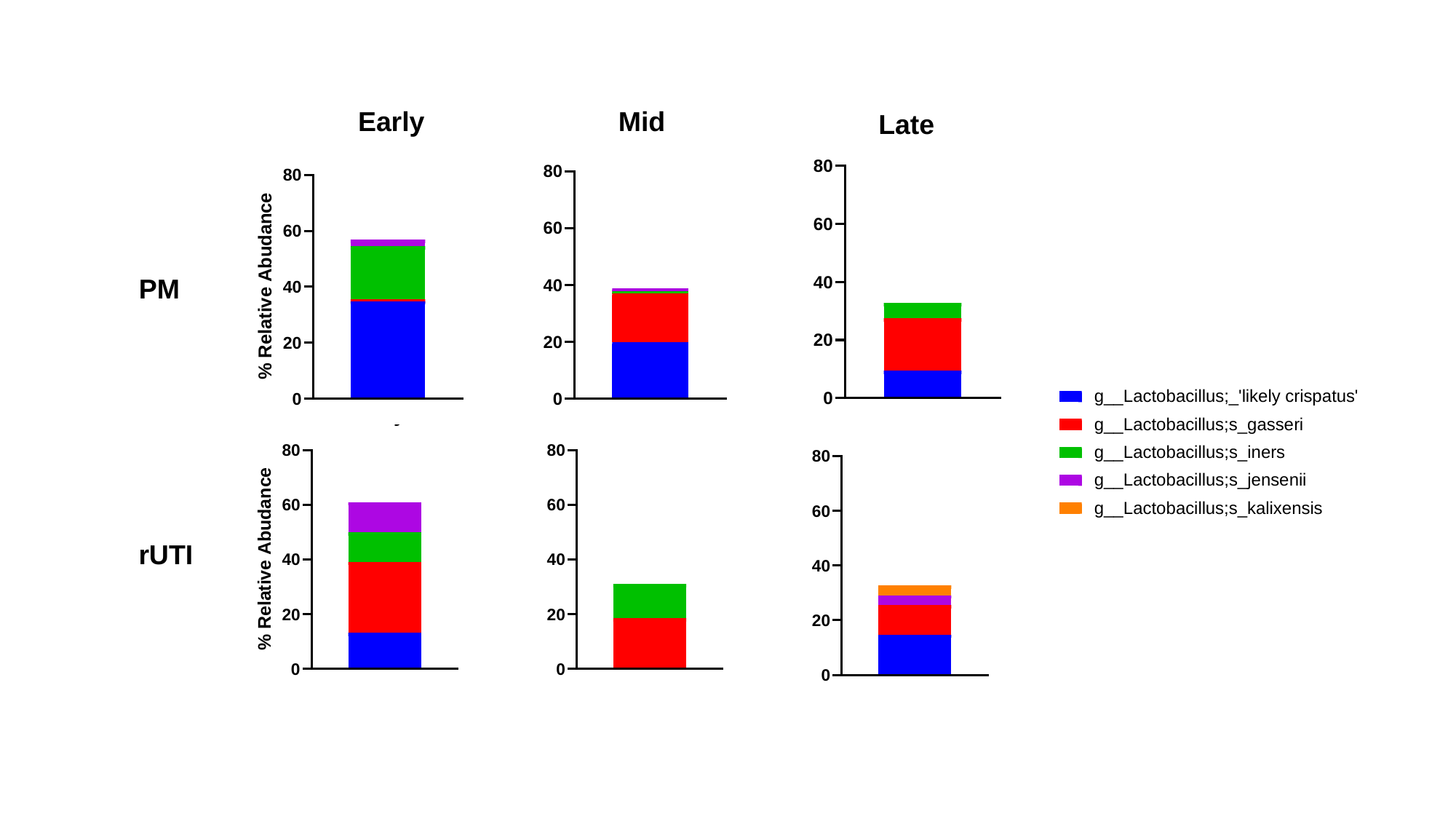

Early
Mid
Late
PM
rUTI

Supplement: SUPPLEMENTARY FIGURE 3 — The stacked barplots show the distribution of Lactobacillus species among age groups post-menopause and between cohorts (controls (PM) versus rUTIs). [file Presentation_3.pptx]

## Slide 1
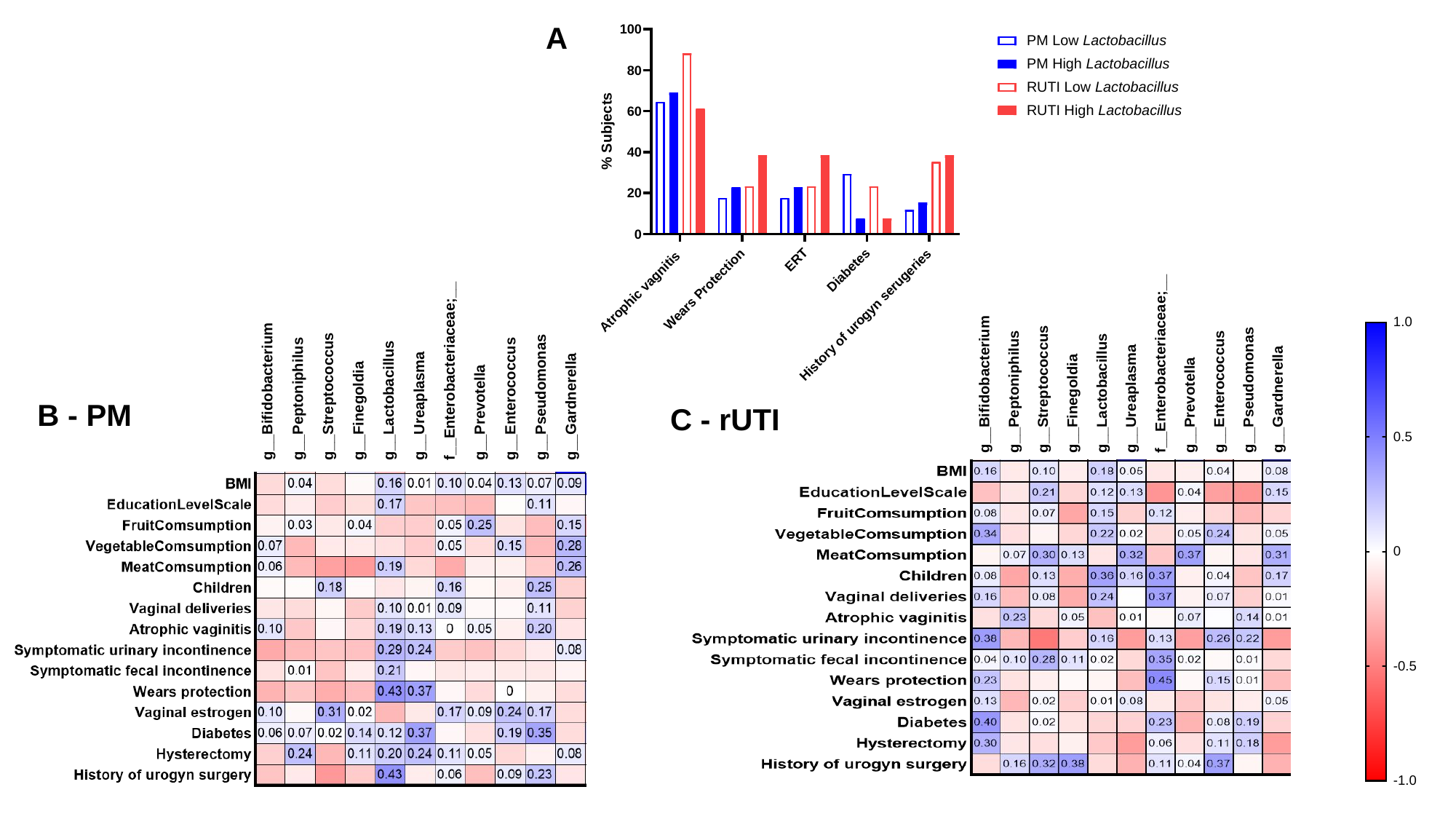

A
B - PM
C - rUTI

Supplement: SUPPLEMENTARY FIGURE 4 — Bars represent the percentage of subjects within each of four pre-defined cohorts who reported a specific clinical characteristic (x-axis). Postmenopausal controls with low Lactobacillus (<50% relative abundance; PM Low Lactobacillus; n = 17), postmenopausal controls with high Lactobacillus (>50%; PM High Lactobacillus; n = 14), rUTI subjects with low Lactobacillus (rUTI Low Lactobacillus; n = 17), and rUTI subjects with high Lactobacillus (rUTI High Lactobacillus; n = 14). The y-axis displays percent of subjects within each of those specific cohorts who have the characteristic listed on the x-axis. ‘Atrophic vaginitis’ includes only symptomatic cases. ERT, Estrogen Replacement Therapy. [file Presentation_4.pptx]
