# Supplementary material for: Vaginal microbiome dysbiosis and a rectal reservoir of uropathogens characterize postmenopausal women with recurrent urinary tract infections: a cross-sectional study
Source: Front Microbiol. 2026 Apr 7;17:1812000. doi: 10.3389/fmicb.2026.1812000 (PMC13096829; doi:10.3389/fmicb.2026.1812000)
Supplement: SUPPLEMENTARY TABLE 2 — Community State Types among Postmenopausal Women with and without recurrent Urinary Tract Infections. This table presents the number of individuals able to be categorized into five different Community State Types (labeled I-V). It compares the distribution of these types between postmenopausal women with recurrent urinary tract infections (Recurrent UTI group) and a Postmenopausal Control group. CST – Community State Type- a classification of the vaginal microbiome; UTI – urinary tract infection. #Total number of CSTs for rUTI were 27. Vaginal microbiomes from four subjects could not be categorized into one of the CSTs and are therefore not reported. [file Table_2.docx]

| **CST** | **Recurrent UTI group^#^** | **Postmenopausal control group** | **P value** |
| --- | --- | --- | --- |
| I | 3 | 6 | p=.790 |
| II | 4 | 6 |  |
| III | 2 | 3 |  |
| IV | 16 | 16 |  |
| V | 2 | 0 |  |

Legend**: TABLE S2. Community State Types among Postmenopausal Women with and without recurrent Urinary Tract Infections.** CST – Community State Type; UTI – urinary tract infection.

#Total number of CSTs for rUTI were 27. Four subject vaginal microbiomes could not be categorized into one of the CSTs.
