# Supplementary material for: Vaginal microbiome dysbiosis and a rectal reservoir of uropathogens characterize postmenopausal women with recurrent urinary tract infections: a cross-sectional study
Source: Front Microbiol. 2026 Apr 7;17:1812000. doi: 10.3389/fmicb.2026.1812000 (PMC13096829; doi:10.3389/fmicb.2026.1812000)
Supplement: SUPPLEMENTARY TABLE 3 — Community State Types among Postmenopausal Women with and without recurrent urinary tract infections separated by time in menopause. Distribution of five distinct Community State Types (CSTs I-V) comparing the Control group (postmenopausal women without recurrent urinary tract infections) and the rUTI group (postmenopausal women with recurrent urinary tract infections). Within each group, participants are further stratified by their time in menopause: Early, Mid, and Late stages. The bottom table provides an aggregated view, showing the Combined counts for both Control and rUTI participants across all categories. Each cell value represents the number of individuals fitting the specified CST and subgroup. Abbreviations: CST: Community State Type, a classification of the vaginal microbiome. rUTI: Recurrent Urinary Tract Infection. $- Group differences were not assessed due to small sample sizes # Due to small sample sizes, to assess the association between years menopause and CST, we dichotomized CST groups as I-III and IV/V, and menopausal years as early and mid/late. This showed a trend toward CST IV/V in mid/late menopause compared to early (65.9% vs. 41.2%, p=0.142). [file Table_3.docx]

| **Postmenopausal Controls^$^** | | | |  | **Recurrent Urinary Tract^$^ Infection** | | | |
| --- | --- | --- | --- | --- | --- | --- | --- | --- |
| **CST** | **Early** | **Mid** | **Late** |  | **CST** | **Early** | **Mid** | **Late** |
| **I** | 3 | 2 | 1 |  | **I** | 1 | 0 | 2 |
| **II** | 0 | 2 | 2 |  | **II** | 3 | 2 | 1 |
| **III** | 2 | 0 | 1 |  | **III** | 1 | 1 | 0 |
| **IV** | 4 | 5 | 7 |  | **IV** | 2 | 6 | 8 |
| **V** | 0 | 0 | 0 |  | **V** | 1 | 0 | 1 |

$- Group differences were not assessed due to small sample sizes

| **Combined Controls and rUTI^#^** | | | | **p Value** |
| --- | --- | --- | --- | --- |
| **CST** | **Early** | **Mid** | **Late** |  |
| **I** | 4 | 2 | 3 | p=0.142 |
| **II** | 3 | 4 | 3 |  |
| **III** | 3 | 1 | 1 |  |
| **IV** | 6 | 11 | 15 |  |
| **V** | 1 | 0 | 1 |  |

Legend: **Table S3. Community State Types among Postmenopausal Women with and without recurrent urinary tract infections separated by time in Menopause.** CST – Community State Types; Early < 10 years menopausal; Mid 10-20 years menopausal; Late > 20 years menopausal

# Due to small sample sizes, to assess the association between years menopause and CST, we dichotomized CST groups as I-III and IV/V, and menopausal years as early and mid/late. This showed a trend toward CST IV/V in mid/late menopause compared to early (65.9% vs. 41.2%, p=0.142).
