# Supplementary material for: Vaginal microbiome dysbiosis and a rectal reservoir of uropathogens characterize postmenopausal women with recurrent urinary tract infections: a cross-sectional study
Source: Front Microbiol. 2026 Apr 7;17:1812000. doi: 10.3389/fmicb.2026.1812000 (PMC13096829; doi:10.3389/fmicb.2026.1812000)
Supplement: Supplementary file 11 [file Supplementary_file_1.docx]

Legend: **Table S1. Uropathogens by Time in Menopause and their Resistance Profile among the Recurrent UTI cohort**.

TMP-SMX- Trimethoprim-Sulfamethaxazole

The values under the antibiotic names are “n” (number of tested samples of that uropathogen showing resistance), then the percentage is based on that resistant / total number of that uropathogen tested for that specific antibiotic.

The Chi-sq listed across the top shows the p-value when comparing the resistance to specific antibiotics between the postmenopausal year groups (<10 years; 10-20 years; >20years).

The Pearsons Chi-Square listed in the left column shows the p-value when comparing the uropathogens across the different postmenopausal year groups (< 10 years;10-20 years;> 20 years)
